# Long-term management of elderly patients with rheumatoid arthritis treated with tocilizumab: comparison of patients over and under 75 years old

**DOI:** 10.3389/fmed.2025.1538170

**Published:** 2025-09-05

**Authors:** Bruno Fautrel, Alain Saraux, Isabelle Idier, Bernard Combe

**Affiliations:** ^1^Sorbonne University-APHP, Rheumatology Department and CRI-IMIDIATE Network, Pitie Salpetriere Hospital, UMRS 1136, Paris, France; ^2^Department of Rheumatology, Université de Bretagne Occidentale, CHU Brest, Inserm UMR 1227 (LBAI), Brest, France; ^3^Medical Department, Chugai Pharma France, Paris La Défense, France; ^4^Department of Rheumatology, Montpellier University, Montpellier, France

**Keywords:** rheumatoid arthritis, tDMARDs, elderly patients, tocilizumab (IL-6R inhibitor), long-term, management

## Abstract

**Background:**

Few real-life long-term data depending on patient age are available in patients starting tocilizumab (TCZ) treatment for rheumatoid arthritis (RA).

**Objectives:**

This study aimed to compare patient characteristics and long-term management with TCZ in RA patients over and under 75 years of age.

**Methods:**

This real-world study was based on secondary data from the French National administrative Healthcare database (SNDS). Eligible adult patients were diagnosed with a RA, and had a first TCZ delivery between 2015/01/01 and 2016/12/31 (index date). Patient data were extracted for the 4 years prior to the index date, and until 2019/12/31. All data were described according to patient age at the index date (<75 and ≥75 years). Therapeutic lines were described from the index date. Maintenance of TCZ therapy was analyzed using the Kaplan Meier method. Adverse events were described using adjusted Hazard Ratios (aHR; reference: age <75 years).

**Results:**

Among the 4,290 analyzed patients (mean age: 57 ± 14 years; female: 77.4%), 3,888 (90.6%) were aged <75 years and 402 (9.4%) were older. Most patients were treated with disease-modifying antirheumatic drugs (DMARDs) for more than 1 year prior to TCZ initiation (<75 years: 83.6%, ≥75 years: 69.9%). At the index date, 48.5% of elderly received SC TCZ (<75 years: 58.1%), 66.2% had TCZ monotherapy (vs. 51.2%), and 83.3% corticosteroids (vs. 76.1%). The median time of TCZ maintenance was similar in both groups [ <75 years: 17.9 months, 95% CI (16.6–19.5); ≥75 years: 14.9 months (11.6–18.6)]. Over follow-up, patients aged at least 75 years experienced more severe and opportunistic infections than younger patients [17.7 vs. 6.9 events per 100 patient-year, aHR: 1.80; 95% CI (1.53–2.13)], acute cardiovascular events [5.6 vs. 1.4; 2.13 (1.60–2.83)], hematological complications [2.0 vs. ≤ 1.0; 1.88 (1.22–2.91)], and deaths [4.7 vs. ≤ 1.0; 3.71 (2.64–5.21)], but no differences were observed for hepatitis, cancer, allergic reaction, and digestive perforation.

**Conclusion:**

This large French nationwide study conducted in patients who initiated TCZ for RA provide reassuring results for patients aged at least 75 years compared to younger patients, in particular regarding treatment maintenance. No new safety signals were identified in elderly patients.

## 1 Introduction

Rheumatoid arthritis (RA) is the most common chronic inflammatory rheumatoid disease affecting about 0.5%−1% of the population and most typically women and elderly (1–3). It is characterized by joint pain and swelling, unpredictable flare-ups leading to progressive joint damage and functional impairment, could include extra-articular manifestations, and increases morbidity and mortality (3–6).

Tocilizumab (TCZ), a humanized anti-IL-6 receptor monoclonal antibody, was marketed in France in 2010 (intravenous-IV form, once-monthly administered at hospital), indicated for adults with moderate-to-severe active RA with inadequate response to at least one DMARD. The subcutaneous (SC) form, once-weekly administered, is also available in France since February 2015.

Older adults are commonly excluded from Research Clinical Trials (RCTs) in RA based on both age limits and other eligibility criteria (7). However, they present with different characteristics at disease onset, including a more equal gender distribution (8). Old age at disease onset was also shown to be associated with worse clinical, radiographic or functional outcomes (9). This contrasts with the use of less aggressive RA treatments, potentially driven by patient comorbidities and polypharmacy, more common in this population (8, 10). Drug maintenance may also be reduced due to safety issues in such populations, more at risk of infections, cancers or cardiovascular events.

Considering the current population aging worldwide (11) including in France (12), the number of elderly patients with RA is growing.

To better know about the RA management in older patients is of particular interest, in particular regarding the treatments provided for RA as well as the drugs' tolerance and safety management knowing that elderly constitute a comorbid and frailer population (10, 13). This appears quite important for TCZ, a drug associated with significant risk of serious infections and digestive perforation. In this context, this study aimed to describe and compare the long-term management with TCZ in RA patients over and under 75 years of age, and tolerance of RA treatments.

## 2 Materials and methods

### 2.1 Study design

This real-world study was based on secondary data from the French National administrative Healthcare database (SNDS) covering 99% of the population living in France (~67 million of people) and collecting individual pseudonymized data from beneficiaries of almost all health insurance schemes (14). Using the SNDS database, eligible adult patients (age ≥18 years) should be diagnosed with a RA (identified using the codes of the 10th revision of the International Classification of Diseases, ICD-10), with a first TCZ delivery (either IV or SC form) between January 1st, 2015 and December 31th, 2016. Patients with at least one TCZ delivery within the last year were excluded.

**Figure 1:**
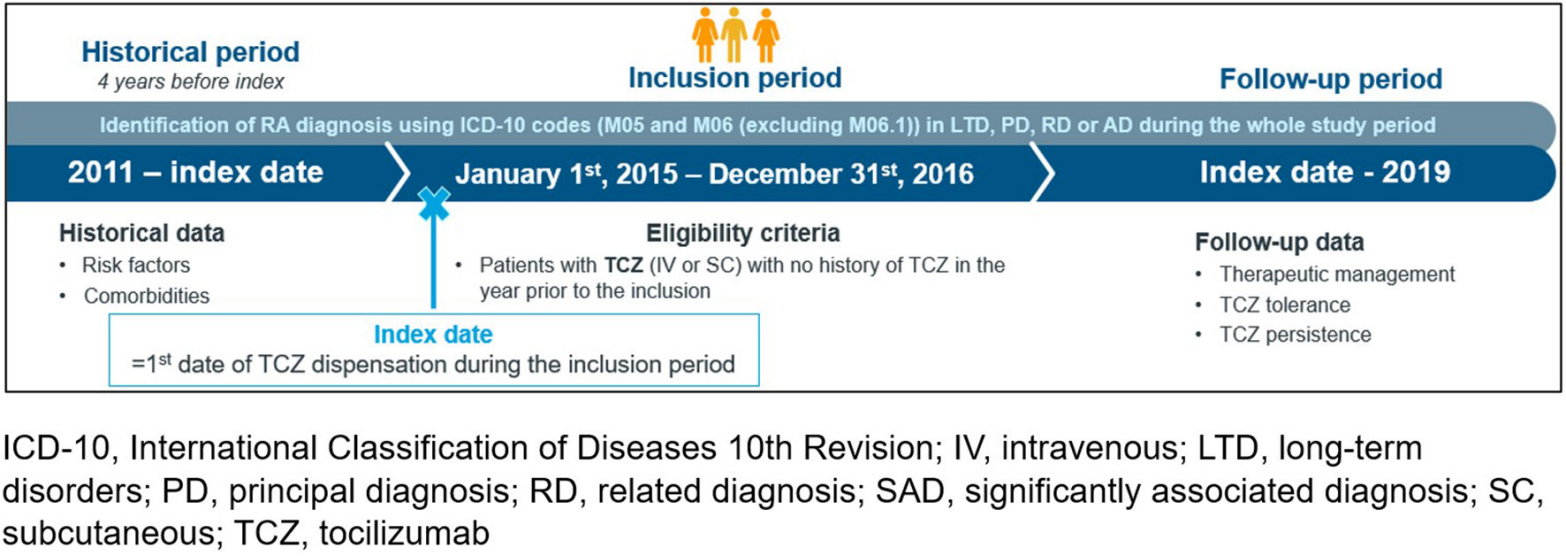
Study design. ICD-10, international classification of diseases 10th revision; IV, intravenous; LTD, long-term disorders; PD, principal diagnosis; RD, related diagnosis; SAD, significantly associated diagnosis; SC, subcutaneous; TCZ, tocilizumab.

Data of eligible patients were extracted from the data base until December 31th, 2019 (i.e., last claim registered in the data base or patient death whatever came first until December 2019) which led to a follow-up duration of 3–5 years after the first TCZ delivery (Figure 1). According to French legislation regarding secondary data use studies, this study was conducted according the MR-004 reference method to ensure patient data confidentiality. It was registered in the French Health Data Hub (No. T24665282020091).

### 2.2 Data collection

In addition to data available during the TCZ initiation-end of follow-up study period, patient data were also extracted from the SNDS data base between January 1st, 2011 and TCZ initiation (index date) in order to provide complementary information about patient characteristics over an at least 4-year historical period. Altogether, the following data were extracted for eligible patients: socio-demographics including patient age, medical history and comorbidities, vaccinal status, previous reimbursed RA treatments, RA characteristics at TCZ initiation, TCZ treatment at initiation (monotherapy or combination with combined RA treatments, form administered, use of corticosteroids, dates of deliveries), subsequent reimbursed RA treatments and dates. The following adverse events of interest were also collected considering the safety profiles of RA treatments (15–17): severe and opportunistic infection, allergic reaction at TCZ administration, hepatitis, hematologic complication, acute cardiovascular event, cancer, digestive perforation, and death. Detailed data extracted from the SNDS data base are provided in the Supplementary Figure 1.

All the useful data of eligible patients were extracted from the SNDS data base on September 4th, 2022.

### 2.3 Study size

All the eligible patients of the SNDS data base covering 99% of the population living in France were included in this study. No sample size calculation was then performed.

### 2.4 Outcomes of interest

The following outcomes of interest were defined: modalities of TCZ use (route of administration, monotherapy or combination, TCZ maintenance), therapeutic sequences after the index date, disease and treatment monitoring according to ongoing guidelines, and adverse events of interest over follow-up.

### 2.5 Statistical methods

All the extracted data were described in the overall analysis population (eligible patients from the SNDS data base) and according to patient age at the index date (<75 and ≥75 years).

Regarding the therapeutic management of RA and treatment sequences, the following “simplified” therapeutic regimens were defined: TCZ only (TCZ MONO), TCZ combined with conventional synthetic disease modifying antirheumatic drug (csDMARD, TCZ COMBO), csDMARD (MONO or COMBO), targeted (t)DMARD MONO (excluding TCZ MONO), and tDMARD COMBO (excluding TCZ COMBO). A therapeutic line was defined by maintained DMARD deliveries without discontinuation, defined as interruption of dispensations for a period superior to 90 days (If an hospitalization occurred during this interruption, the hospitalization duration was added to the exposition period) or permanent treatment discontinuation, switch to another RA treatment or addition of a conventional synthetic (cs)DMARD. Accordingly, the first line of treatment of each patient was the one starting at the index date by the initiation of TCZ. A change defined a new line of RA treatments.

Maintenance of TCZ therapy was described by age groups using the Kaplan Meier method. The proportion of patients with at least one adverse event of interest was described in 100 patient-year. Comparisons between age groups were performed using an adjusted Cox proportional-hazard model with time dependent variables in order to consider the change in lines of treatments (only the first event was considered). Adjustment were performed on the following covariates: age, sex, severe infection, cardiovascular (acute events + disease grouped), cancer, lung diseases, gastrointestinal diseases, liver disease, use of anti-hypertensive treatments, end stage renal disease, diabetes, dyslipidemia treated with statins, use of anti-depressive treatments, morbid obesity, formulation at index date (IV/SC), initiation of DMARDs at index date and daily dose of glucocorticoids (prednisone equivalent) at initiation. Adjusted Hazard Ratios (aHRs) were provided with associated 95% confidence intervals (CI), the group of patients aged <75 years being the statistical reference.

The SNDS medico-administrative database being known to be highly comprehensive and accurate for the specific variables identified, no missing data were anticipated, and no handling of missing data was performed.

Analysis was performed, using SAS^®^ software (SAS Institute, North Carolina, USA), version 9.4.

## 3 Results

### 3.1 Populations

**Figure 2:**
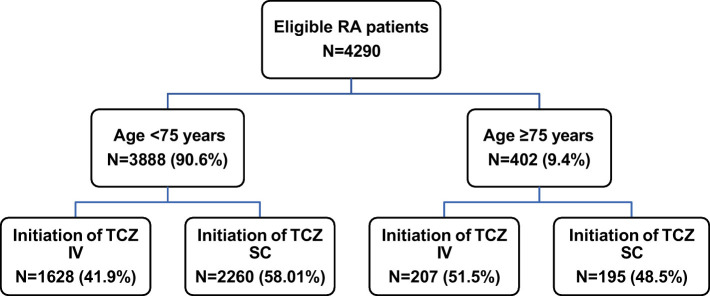
Patient flowchart. IV, intravenous; RA, rheumatoid arthritis; SC, subcutaneous; TCZ tocilizumab.

Between January 1st, 2015, and December 31st, 2016, a total of 4,290 eligible RA patients initiated TCZ treatment and were then included in this study. Among them, 3,888 patients (90.6%) were aged <75 years and 402 patients (9.4%) were older (Figure 2).

Among the 4,290 analyzed patients, the yearly numbers of those followed in this study were 4,290 (100%), 4,240 (98.9%), 4,180 (97.4%), 4,076 (95.0%), and 1,814 (42.3%) at year 1, 2, 3, 4, and 5, respectively.

### 3.2 Patient and disease characteristics

Patient characteristics are detailed in Table 1. Overall, the mean age of patients was 56.8 ± 13.9 years. Female patients were in the majority (77.4%) with no differences according to patient groups. Globally, patients aged at least 75 years presented with more medical history and comorbidities than younger patients, within the 4 years prior to TCZ initiation. In particular, they suffered from cardiovascular diseases more frequently (15.9 vs. 5.2%), experienced more acute cardiovascular events (13.4 vs. 4.7%) or severe infections (32.3 vs. 19.4%). They also took antihypertensive treatments and statins more often (73.4 vs. 35.2% and 42.8 vs. 18.0%, respectively). Of note, the influenza and pneumococcal vaccine coverages were low, even in older patients (55.5 and 42.0%, respectively).

**Table 1:** Characteristics of patients with rheumatoid arthritis at tocilizumab initiation.

**Characteristics**	**Total**	<**75 years**	≥**75 years**
	***N*** = **4,290**	***N*** = **3,888**	***N*** = **402**
**Age, mean ± SD**	**56.8 ± 13.9**	**54.5 ± 12.4**	**79.4 ± 3.8**
**Gender**, ***n*** **(%)**			
Female patients	3,321 (77.4)	3,009 (77.4)	312 (77.6)
**Medical history within the last 4 years**, ***n*** **(%)**			
Severe infection	884 (20.6)	754 (19.4)	130 (32.3)
Acute cardiovascular event	237 (5.5)	183 (4.7)	54 (13.4)
Cancer	125 (2.9)	95 (2.4)	30 (7.5)
Cardiovascular diseases	265 (6.2)	201 (5.2)	64 (15.9)
Lung disease	108 (2.5)	87 (2.2)	21 (5.2)
Other chronic pulmonary diseases	999 (23.3)	878 (22.6)	121 (30.1)
Gastrointestinal diseases (diverticulosis)	178 (4.1)	142 (3.7)	36 (9.0)
Liver disease	161 (3.8)	150 (3.9)	11 (2.7)
Use of anti-hypertensive treatments	1,663 (38.8)	1,368 (35.2)	295 (73.4)
End stage renal disease	≤ 10 (≤ 0.2)^*^	≤ 10 (≤ 0.3)^*^	–
Diabetes	493 (11.5)	422 (10.9)	71 (17.7)
Use of statins	871 (20.3)	699 (18.0)	172 (42.8)
Use of anti-depressive treatments	940 (21.9)	843 (21.7)	97 (24.1)
Morbid obesity	557 (13.0)	509 (13.1)	48 (11.9)
**Vaccination**, ***n*** **(%)**			
Influenza vaccination *within the last year*	1,017 (23.7)	794 (20.4)	223 (55.5)
Pneumococcal vaccination *within the last 4 years*	2,263 (52.8)	2,094 (53.9)	169 (42.0)

^*^In accordance with the General Data Protection Regulation (GDPR), all results for *n* ≤ 10 patients have not been displayed.

### 3.3 Treatment strategies at tocilizumab initiation and during patient follow-up

Most patients were already treated with DMARDs for more than 1 year prior to TCZ initiation (<75 years: 83.6%, ≥75 years: 69.9%); tDMARDs were given in 41.1 and 33.8% of patients, respectively. At TCZ initiation, the majority of patients (*n* = 2,455, 57.2%) received SC TCZ but this proportion was lower in patients aged at least 75 years (48.5 vs. 58.1% of patients under 75; Table 2 and Figure 2). Older patients received TCZ monotherapy more often (66.2 vs. 51.2%). In both patient groups, methotrexate was the most frequently combined treatment when TCZ was given in combination (in 73.5 and 77.8% of the cases, respectively). The majority of patients were receiving corticosteroids (83.3 and 76.1%; respectively).

**Table 2:** Tocilizumab therapy at treatment initiation.

**Tocilizumab therapy**	**Total**	<**75 years**	≥**75 years**
	***N*** = **4,290**	***N*** = **3,888**	***N*** = **402**
**At tocilizumab initiation**, ***n*** **(%)**			
TCZ SC	2,455 (57.2)	2,260 (58.1)	195 (48.5)
TCZ IV	1,835 (42.8)	1,628 (41.9)	207 (51.5)
TCZ monotherapy	2,255 (52.6)	1,989 (51.2)	266 (66.2)
TCZ in combination	2,035 (47.4)	1,899 (48.8)	136 (33.8)
With MTX	1,568 (77.1)	1,478 (77.8)	100 (73.5)
With LFN	295 (14.5)	275 (14.5)	20 (14.7)
With HCQ	50 (2.5)	44 (2.3)	≤ 10 (≤ 7.4)^*^
With MTX and HCQ	48 (2.4)	45 (2.4)	≤ 10 (≤ 7.4)^*^
With SZS	44 (2.2)	–	–
With MTX and SZS	11 (0.5)	≤ 10 (≤ 0.5)^*^	≤ 10 (≤ 7.4)^*^
With HCQ and SZS	≤ 10 (≤ 0.5)^*^	≤ 10 (≤ 0.5)^*^	≤ 10 (≤ 7.4)^*^
With MTX, HCQ and SZS	≤ 10 (≤ 0.5)^*^	≤ 10 (≤ 0.5)^*^	–
**DMARD initiation within the last 12 months**, ***n*** **(%)**			
Yes	760 (17.7)	639 (16.4)	121 (30.1)
No	3,530 (82.3)	3,249 (83.6)	281 (69.9)
Previous use of tDMARD	1,735 (40.4)	1,599 (41.1)	136 (33.8)
In monotherapy	1,154 (66.5)	1,046 (65.4)	108 (79.4)
In combination	581 (33.5)	553 (34.6)	28 (20.6)

DMARD, disease modifying antirheumatic drug; HCQ, hydroxychloroquine; IV, intravenous; LFN, leflunomide; MTX, methotrexate; SZS, sulfasalazine; SC, sub-cutaneous; TCZ, tocilizumab; tDMARD, targeted DMARD.

^*^In accordance with the General Data Protection Regulation (GDPR), all results for *n* ≤ 10 patients have not been displayed.

**Figure 3:**
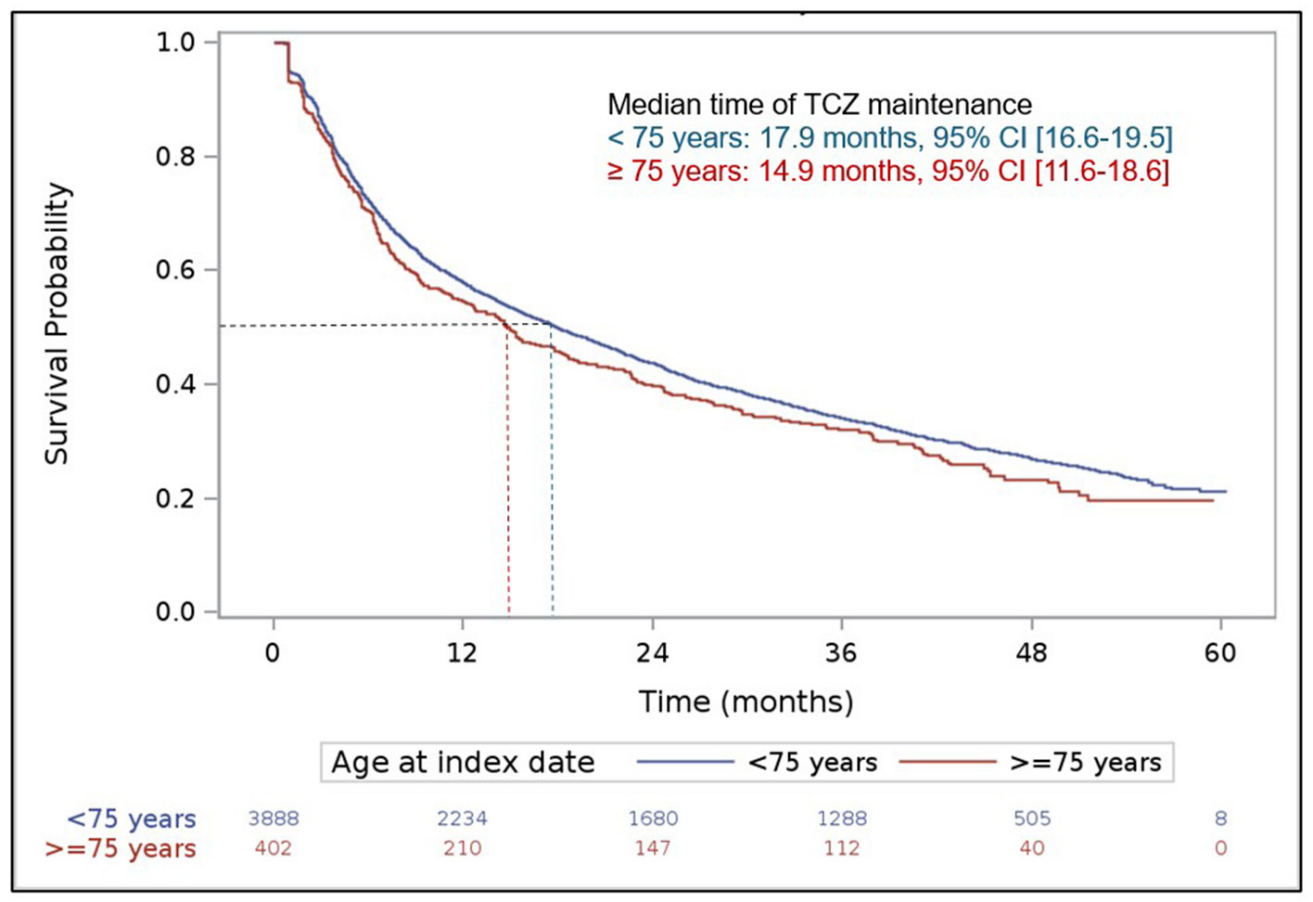
Maintenance of tocilizumab treatment over patient follow-up. Kaplan Meier curve. The index date was defined by the date of the first delivery of tocilizumab.

During patient follow-up, 3,248 (75.7% of the 4,290 analyzed patients) received a second line of treatment after the index date (i.e., treatment after a first line of TCZ) and 2,102 (49.0%) a third therapeutic line. Over this period, patients aged at least 75 years were more numerous to maintain their first line of treatment (TCZ MONO or COMBO) without any change compared to younger patients (21.9 vs. 18.0%), respectively. In patients with treatment change after the first line following the index date, a temporary discontinuation (>90 days) was more frequently reported in elderly patients (27.6 vs. 18.3%) while removals of one drug of the TCZ COMBO regimen and switches to other RA treatments were less frequent (21.6 vs. 28.5% and 22.6 vs. 27.1%, respectively). The median duration of the two first therapeutic lines after the index date was close in the two populations and tended to decrease overtime (first line: 10.3 vs. 9.0 months in patients aged at least 75 years, second line: 8.2 vs. 7.7 months, respectively). The respective median durations of the third line of treatment after the index date were 6.2 and 8.4 months. Using the Kaplan–Meier method, the median time of TCZ maintenance was not statistically different in both patient groups [ <75 years: 17.9 months,≥75 years: 14.9 months, with overlapping 95% ICs: (16.6–19.5) and (11.6–18.6), respectively; Figure 3], as well as the respective treatment maintenance rates at 1, 2, 3, and 4 years after TCZ initiation (<75 years: 57.8, 43.7, 34.1 and 26.9%, respectively; ≥75 years: 54.7, 42.2, 32.0 and 23.4%, respectively).

**Figure 4:**
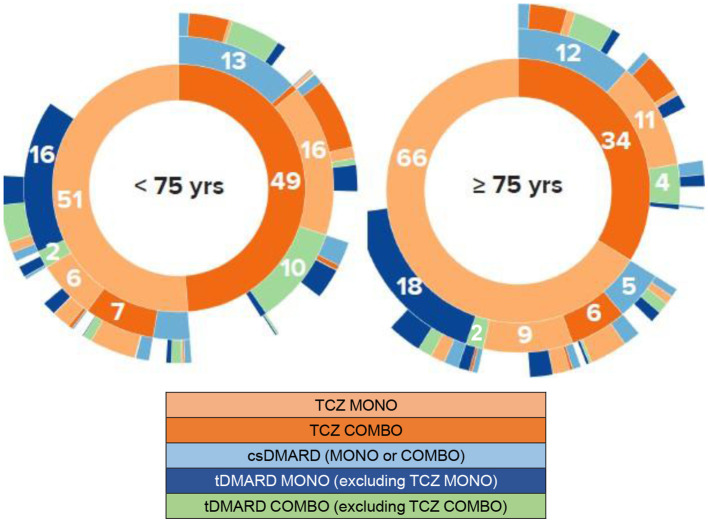
Chronological lines of treatment from tocilizumab initiation. Sunburst representations. COMBO, combination; csDMARD, conventional synthetic disease modifying antirheumatic drug; MONO, monotherapy; tDMARD, targeted DMARD; TCZ, tocilizumab; yrs, years. These sunburst diagrams allow to visualize the successive treatments of patients for each of the three treatment lines being depicted by a concentric circle (hierarchical data), The circle in the center represents the first treatment line at tocilizumab initiation (i.e., line 1, inner-most ring in the sunburst plot), with the hierarchy moving outward from the center. A segment of the inner circle bears a hierarchical relationship to those segments of the outer circle which lie within the angular sweep of the parent segment. Numbers represent the percentage of patients receiving/switching to the specific treatment for each line. For example, on the line 1, 66% of the 402 patients aged at least 75 years were treated with TCZ MONO and 34% with TCZ COMBO. On the line 2 (second circle), considering the patients with TCZ MONO on the line 1, 9% of all patients maintained TCZ MONO,6% received TCZ COMBO,5% had csDMARDs MONO or COMBO, 2% tDMARD COMBO, and 18% tDMARD MONO.

Treatment regimen given for RA at each of the first three lines of treatment after the index date are represented graphically in Figure 4 according to patient age. In addition, older patients received corticosteroids more often than other patients whatever the therapeutic line after the index date was (first treatment line: 83.3 vs. 76.1%; second line: 86.0 vs. 70.1%; third line: 79.1 vs. 66.3%).

### 3.4 Compliance to recommendations under treatment for rheumatoid arthritis

One year after TCZ initiation, most of the recommended blood tests (inflammatory tests, complete blood count, and liver tests) were performed in at least 90% of patients, whatever the age group was (Table 3). Lipid tests were carried out in a minority of patients at that time but more frequently in older patients (≥75 years: 25.1%: <75 years: 10.2%). Regarding the management of osteoporosis, elderly patients also received calcium/vitamin D and/or anti-osteoporotic drugs more often than other patients (74.4 vs. 57.9% and 25.1 vs. 10.2%, respectively).

**Table 3:** Compliance to recommendations under RA treatment, 1 year after tocilizumab initiation.

**Parameter, *n* (%)**	**Total**	** < 75 years**	**≥75 years**
	***N* = 4,290**	***N* = 3,888**	***N* = 402**
**Blood tests**			
CRP	4,110 (100.0)	3,722 (100.0)	388 (100.0)
Complete blood count	4,149 (100.0)	3,755 (100.0)	394 (100.0)
Transaminases	4,104 (95.7)	3,720 (95.7)	384 (95.5)
Lipid tests	443 (10.3)	408 (10.5)	35 (8.7)
**Osteoporosis management**			
Use of anti-osteoporotic drugs	498 (11.6)	397 (10.2)	101 (25.1)
Use of calcium/vitamin D	2,552 (59.5)	2,253 (57.9)	299 (74.4)

CRP, C-reactive protein; ESR, erythrocyte sedimentation rate, RA, rheumatoid arthritis.

### 3.5 Safety

Overall, and regardless of treatment delivered, the most reported AEs of interest during patient follow-up were severe and opportunistic infections (*n* = 7.7/100 patient-year), acute cardiovascular events (*n* = 1.7), and digestive perforation (*n* = 1.6; Table 4). No allergic reactions following TCZ administration were reported. Patients aged at least 75 years experienced more severe and opportunistic infections than younger patients [17.7 vs. 6.9 events per 100 patient-year, aHR: 1.80; 95% CI (1.53–2.13)], acute cardiovascular events [5.6 vs. 1.4; 2.13 (1.60–2.83)], and hematological complications [2.0 vs. ≤ 1.0; 1.88 (1.22–2.91)]. They also deceased more frequently during follow-up [4.7 vs. ≤ 1.0; 3.71 (2.64–5.21)]. However, no statistical differences between groups (*p* > 0.05) were observed for hepatitis, cancer, allergic reactions, and digestive perforation, with limited incidence rates [between 0 (allergic reactions) and 1.6 (digestive perforation) per 100 patient-year]. Regardless of age, there were no differences in the number of events per 100 patients-years for severe and opportunistic infection, acute cardiovascular event, hepatitis, cancer, digestive perforation between patients receiving TCZ MONO or COMBO, or other tDMARD MONO or COMBO. Hematological complications were slightly more frequent in TCZ groups when compared to other tDMARD groups (Supplementary Table 1).

**Table 4:** Adverse events of interest (AEs) according to patient age, regardless of treatment delivered.

**AEs of interest**	**Incidence of AEs of interest (per 100 patient-year)**, ***n***			**aHR [95% CI] (ref: <75 years)**
	**Total *N* = 4,290**	** <75 years *N* = 3,888**	**≥75 years *N* = 402**	
Severe and opportunistic infection	7.7	6.9	17.7	1.80 [1.53–2.13]
Acute cardiovascular event^*^	1.7	1.4	5.6	2.13 [1.60–2.83]
Digestive perforation	1.6	1.5	3.1	1.32 [0.92–1.88]
Hematologic complication^**^	≤ 1.0	≤ 1.0	2.0	1.88 [1.22–2.91]
Cancer	≤ 1.0	≤ 1.0	1.2	1.06 [0.63–1.79]
Hepatitis	≤ 1.0	≤ 1.0	≤ 1.0	1.20 [0.36–3.99]
Allergic reaction to treatment perfusion/injection	0	0	0	/
Death	≤ 1.0	≤ 1.0	4.7	3.71 [2.64–5.21]

aHR, adjusted hazard ratio (≥75 vs. <75 years); CI, confidence interval; ref, reference.

^*^Including myocardial infarction, stroke and low limb acute ischemia.

^**^Including neutropenia leading to hospitalization.

Only first event was considered. In accordance with the General Data Protection Regulation (GDPR), all results for n ≤ 10 patients have not been displayed.

## 4 Discussion

### 4.1 Discussion on key study results

This real-world French nationwide study allowed to compare the long-term management of RA patients aged at least 75 years and who initiated TCZ with younger patients. Indeed, as reported in France on the basis of 2010–2019 data from the SNDS, older patients are those most affected by the disease, whatever their treatment is (mean age of RA patients in 2019: 66 ± 17 years) (3).

In our study, the mean age of RA patients who initiated TCZ between 2015 and 2016 was lower (57 ± 14 years). However, it was similar to those observed in the previous non- interventional PEPS, SPARE-1, and ACT-SOLO, studies conducted when only the IV form of TCZ was commercially available in France (56 or 57 years) (18–20). As expected (10, 13), older RA patients presented with more medical history and comorbidities than patients aged <75 years. It is probably the reason why they received TCZ monotherapy more frequently at treatment initiation as previously reported in elderly patients (18, 21–23), in a real-life setting. In addition, they received the SC form of TCZ more often than younger patients. In the other hand, more older patients were treated with corticosteroids, in line with previous findings (21, 24).

Over the study period (3–5 years after the first TCZ delivery), patients aged at least 75 years were more likely to maintain their first line of RA treatment, that is TCZ in monotherapy or combination, without any change, or to temporarily discontinue RA treatment (>90 days) during their first three therapeutic lines (first line with TCZ and next two lines). These interruptions may be notably related to the adverse events they experienced more often than younger patients (severe and opportunistic infections, acute cardiovascular events, or hematological complications including neutropenia leading to hospitalization). It should be noted that elderly people are already a comorbid population (10, 13), with an additional potential higher risk for infections under biologic disease- modifying antirheumatic drugs (bDMARDs) due to the decline of the immune system over the years (25). However, no differences between patient age groups were observed for other events of interest (hepatitis, cancer, allergic reaction at TCZ administration, and digestive perforation) with limited incidence rates. By comparison to previous reports, infections were also the most reported adverse events in patients treated with TCZ (19, 20), with a similar risk of hospitalized infections between TCZ and other biological or targeted bDMARDs (26). However, according to the literature, the rates of adverse events assessed as related to RA therapies were similar in elderly compared to younger RA patients (8, 25) as well as the rates of adverse events leading to withdrawal (21, 24). In addition, as expected, older patients had a higher risk to die over the study period compared to younger patients. As the causes of death were not available in the database, no link could be made with the events of interest.

Despite these indicators of frailty in older RA patients, no differences were observed in terms of TCZ maintenance according to patient age in this study. This result is in accordance with previous real-life data comparing elderly and younger patients (21, 24). Altogether, our results showed that the long-term therapeutic management of older patients who initiated TCZ for RA was close to that of younger patients, even they were frailer.

According to recommendations regarding patient follow-up under RA treatments (27), blood tests (complete blood count and transaminases in particular) were performed in almost all of the patients 1 year after TCZ initiation whatever their age group was. In addition, prophylaxis and treatment of osteoporosis were much more frequently provided in elderly suggesting that the frequent long-term use of corticosteroids in older RA patients was notably taken into account in addition to their advanced age, in accordance to recommendations (28). Overall, our results are aligned with a recent systematic review performed to establish clinical practice recommendations for the use of DMARDs and GCs in older people (29).

### 4.2 Limitations

Our study has some limitations, mainly due to the use of data available in the SNDS. In particular, treatment durations, analyzed on the basis of the dates of deliveries, could be overestimated as patient treatment compliance could not be taken into account. In addition, the definition of treatment discontinuation (> 90 days) could lead to an underestimation of the number of treatment lines over patient follow-up. Additionally, the general data protection regulation (GDPR) limited the precision of safety data included in the database, compared to RCTs or prospective cohorts. Finally, as known potential factors impacting RA management are not available in the database (RA disease activity and severity, laboratory test results and reasons for treatment discontinuation), it is not possible to fully explain RA care according to patient groups. However, as these biases are independent of the groups of patients defined by their age at TCZ initiation, we assume that our results comparing data between patient groups remain robust.

No comparison of maintenance and safety outcomes was done in the study between TCZ and other bDMARDs, which could be seen as a limitation. This was not the aim of the study and thus, not planned in the approved statistical analysis plan.

## 5 Conclusion

This large French nationwide study conducted in RA patients who initiated TCZ treatment provides detailed information on their long-term therapeutic management in the real-life setting. Reassuring results are shown for patients aged at least 75 years compared to younger patients, in particular regarding treatment maintenance in routine medical practice. No new safety signals were identified in elderly patients. However, a personalized therapeutic approach has to be adopted in routine medical practice to take into account the risk profiles of elderly RA patients, notably risk factors for infections and cardiovascular events.

## Data Availability

The data are the property of the Health Data Hub, and can be made available only upon formal and reasonable request to the corresponding author.
