# Supplementary figures and images for: Long-term management of elderly patients with rheumatoid arthritis treated with tocilizumab: comparison of patients over and under 75 years old
Source: Front Med (Lausanne). 2025 Sep 5;12:1538170. doi: 10.3389/fmed.2025.1538170 (PMC12447570; doi:10.3389/fmed.2025.1538170)

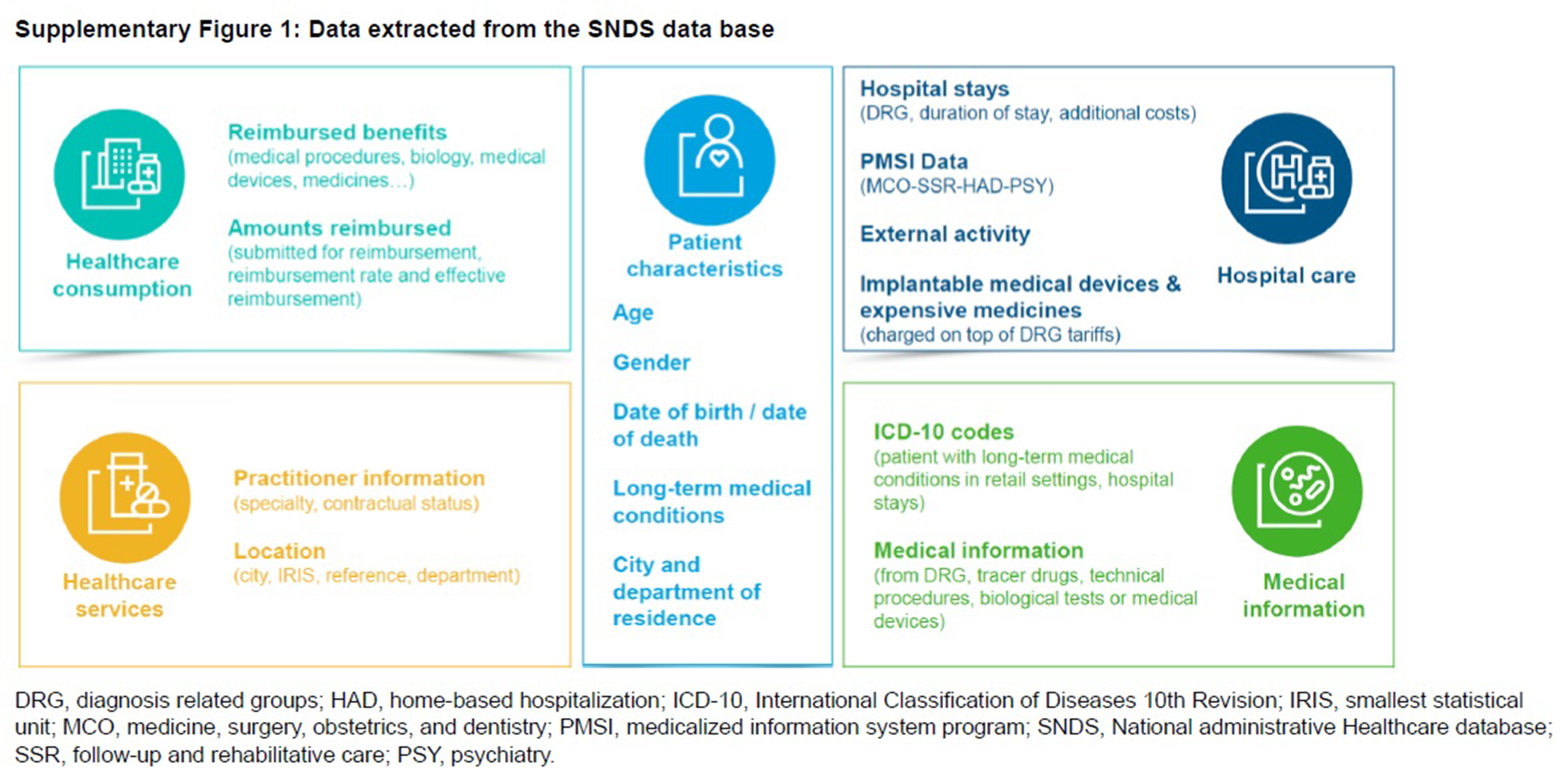

Supplement: Supplementary file 1 [file Image_1.jpeg]
