# Supplementary material for: Long-term management of elderly patients with rheumatoid arthritis treated with tocilizumab: comparison of patients over and under 75 years old
Source: Front Med (Lausanne). 2025 Sep 5;12:1538170. doi: 10.3389/fmed.2025.1538170 (PMC12447570; doi:10.3389/fmed.2025.1538170)
Supplement: Supplementary file 2 [file Table_1.docx]

**Supplementary Table 1: Adverse events of interest (AEs) according to the** t**DMARD delivered, regardless of patient age**

| **AEs of interest** | **Incidence of AEs of interest [95% CI] (per 100 person-year)** | | | |
| --- | --- | --- | --- | --- |
|  | **TCZ MONO**  **(n=4149)** | **TCZ COMBO**  **(n=3270)** | **tDMARD MONO (excluding TCZ MONO)**  **(n=2139)** | **tDMARD COMBO (excluding TCZ COMBO)**  **(n=1801)** |
| **Severe and opportunistic infection** | 6.5 [5.7-7.2] | 6.5 [5.6-7.4] | 7.0 [5.8-8.2] | 5.5 [4.3-6.6] |
| **Acute cardiovascular event** | 1.5 [1.1-1.8} | 1.1 [0.7-1.4] | 1.5 [1.0-2.0] | 0.8 [0.4-1.2] |
| **Digestive perforation** | 1.8 [1.4-2.1] | 1.5 [1.1-1.9] | 1.6 [1.1-2.2] | 1.1 [0.6-1.6] |
| **Hematologic complication** | 0.8 [0.5-1.0] | 1.3 [1.0-1.7] | 0.2 [0.0-0.4] | 0.5 [0.2-0.9] |
| **Cancer** | 0.5 [0.3-0.6] | 0.4 [0.2-0.6] | 0.7 [0.3-1.1] | 0.8 [0.4-1.3] |
| **Hepatitis** | 0.2 [0.1-0.3] | 0.2 [0.0-0.3] | 0.2 [0.0-0.4] | 0.1 [0.0-0.2] |
| **Allergic reaction at perfusion/injection** | 0.0 [0.0-0.1] | 0.0 [0.0-0.1] | 0.0 [0.0-0.0] | 0.0 [NA] |
| **Death** | 0.3 [0.1-0.4] | 0.2 [0.0-0.3] | 0.5 [0.2-0.8] | 0.2 [0.2-1.0] |

CI, confidence interval; COMBO, combination; MONO, monotherapy; NA, not applicable; tDMARD, targeted disease modifying antirheumatic drug; TCZ, tocilizumab
